# Normalisation of Multicondition cDNA Macroarray Data

**DOI:** 10.1155/2007/90578

**Published:** 2007-04-22

**Authors:** Nicola L. Dawes, Jarka Glassey

**Affiliations:** School of Chemical Engineering and Advanced Materials, Merz Court, University of Newcastle upon Tyne, Newcastle upon Tyne, NE1 7RU, UK

## Abstract

*Background*. Normalisation is a critical step in obtaining meaningful information from the high-dimensional DNA array data. This is particularly important when complex biological hypotheses/questions, such a functional analysis and regulatory interactions within biological systems, are investigated. A nonparametric, intensity-dependent normalisation method based on global identification of self-consistent set (SCS) of genes is proposed here for such systems. *Results*. The SCS normalisation is introduced and its behaviour demonstrated for a range of user-defined parameters affecting its performance. It is compared to a standard global normalisation method in terms of noise reduction and signal retention. *Conclusions*. The SCS normalisation results using 16 macroarray data sets from a *Bacillus subtilis* experiment confirm that the method is capable of reducing undesirable experimental variation whilst retaining important biological information. The ease and speed of implementation mean that this method can be easily adapted to other multicondition time/strain series single colour array data.

## 1. BACKGROUND

DNA array-based approaches have been widely applied for gene expression studies in many research areas from functional genomics to biomedical applications. As these studies become more rigorous, they require the use of multiple DNA arrays and normalisation is a key issue in the analysis of the resulting data. Indeed, as Edwards [1] claims “Normalisation has profound effects on subsequent analysis, irrespective of the methodology used. Failure to normalize appropriately will generally lead to misleading conclusions.” An effective normalisation technique is one that reduces experimental variation or biases (noise) without affecting the measurement of the biological variation (signal). There are a number of well-documented normalisation techniques ranging from simple scaling methods to more complex statistical approaches [2–4]. “Global” scaling methods are suitable for data sets where relatively few genes are expected to change between conditions and global array statistics such as median/mean expression levels can be used. Statistical models require a good level of replication of experiments in order to give acceptable results [5].

**Figure 1:**
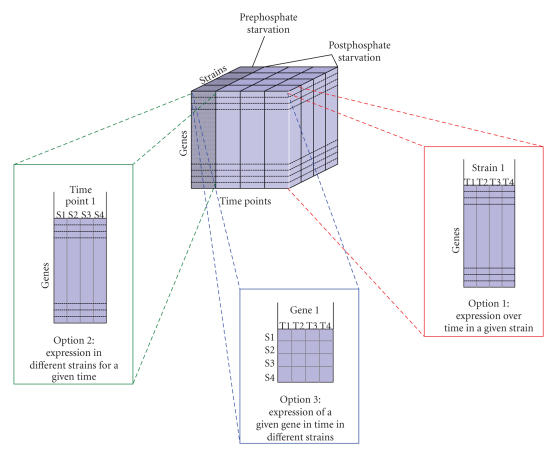
Three-dimensional structure of the *B. subtilis* gene expression data set. Graphical representation of the experimental data available for analysis illustrating the complex relationships and the optional simplified biological hypothesis that can be investigated.

For some systems, normalisation is built into the experimental design using specific software for the DNA array system, such as the Affymetrix system. Some DNA array experiments, however, produce data that is not so easily normalised. This paper focuses on the normalisation of multicondition time series gene expression data, generated using one-colour membrane macroarrays. For details of the experiment, see Materials and Methods. The data set consists of data from 16 hybridisation experiments, using four strains of the organism *Bacillus subtilis* grown in phosphate-limiting conditions as shown in Figure 1. The four strains are referred to as “wildtype” (*B. subtilis * 168 WT), “sigB” (*sigB*-null mutant 168-ML6), “phoR” (*phoR*-null mutant 168-PR), and “double mutant” (*sigB*-null, *phoR*-null mutant 168-ML6PR). Samples were taken from each strain at 4 time points across the transition to phosphate limitation.

In the resulting data set, the expression of many genes was affected by the phosphate-limiting conditions and modified in the mutant strains. It is theoretically possible to analyse this data set using some traditional methods of normalisation in order to answer simplified biological hypotheses represented in Figure 1 as Options 1, 2, and 3. However, in order to investigate the mechanisms of interaction between general and specific phosphate-stress response regulons, it is essential to use all the data and normalise it appropriately to avoid loss of important biological variation (signal).

Currently, the scientific literature mainly reports on more straightforward investigations of either single time points from a variety of strains/conditions or time profile of gene expression from a single strain using traditional methods of normalisation. However, functional genomics in particular will require an alternative approach to both experimental design and data analysis and hence novel normalisation methods, such as the one proposed here, will become more appropriate.

Depending on the array construction, the application of statistical modelling methods, which rely more heavily on data replication [6], may be limited. Also, most other new normalisation methods have been developed using data from two-colour arrays [7, 8] and in some cases these methods are not suitable for one-colour array data. A number of these normalisation methods are based on the identification of a group of genes deemed to be invariant [9] often between the two-colour channels on a given array [4]. This can only be translated to the analysis of one-colour array data by comparing all the arrays in the experiment to one array, taken as the baseline array. It is not possible to choose a suitable baseline array in the *B. subtilis* data set due to biological variability between the strains as well as across the time trajectory within each strain as a result of growth and phosphate starvation. Therefore a new normalisation method, which does not require a selection of a baseline array, is proposed to identify a set of invariant genes globally, across all the arrays simultaneously.

This manuscript sets out in detail the proposed normalisation method and investigates the sensitivity of the algorithm to two critical parameters. Ideally, the benefits of a new normalisation method would be judged by the improvements in clustering the data compared to nonnormalised data (or data normalised using alternative normalisation methods). However, this requires knowledge of expected composition of clusters in order to assess whether correct clusters are identified. In this particular application, gaining such knowledge is indeed the overall aim of the experimental work and hence it is not available at this stage. Thus a comparison in terms of a simple nonparametric scoring method [10] (referred to here as the “Park” score) is used to highlight the improvements in identifying differentially expressed genes (known to be under the control of the pho regulon) from the normalised data.

Given the problems in quantifying the benefits of the new normalisation procedure on the biological system studied, it would be desirable to compare this procedure with standard normalisation methods used on data publicly available. However, cDNA array data currently publicly available does not have the same structure (gene expression measured for a number of conditions over a number of time points following the same experimental protocol) and hence this normalisation procedure could not be applied to such data.

The main problem with identifying the best (or most appropriate) normalisation method is the lack of a “gold standard”—a validation set that can reflect the complexity of real data [4]. Thus one of two approaches usually employed to compare different normalisation methods or to introduce a new one is the ability to reduce noise in the data and the ability to retain biological signal. A number of approaches were used to asses the noise reduction, such as MA diagnostic plots used for two-colour cDNA microarrays [11] or coefficient of variation [4]. Unfortunately, the data investigated in this work does not contain technical replicates other than the duplicate spots on the same array. These cannot be used for the purposes of noise reduction assessment although they were used in MA plots to establish the reliability of each of the gene expression values as well as to check whether the proposed normalisation introduces any undesirable artefacts into the data. Thus alternative means of demonstrating noise reduction and a more plausible biological explanation of the expression data is provided in this manuscript. The ability to retain biological signal is demonstrated through the changes in the detection of differentially expressed genes.

## 2. RESULTS

### 2.1. Self-consistent set normalisation

The SCS normalisation process can be seen as a series of filters implemented in the SCS algorithm described in the Materials and Methods section. A gene must pass all the filters to be classed as “self-consistent.” The filters are based on the absolute difference in rank position of a gene's contribution to the total expression on a single array between the arrays representing corresponding time points for each strain. For each time point, comparisons were made between wildtype and sigB; wildtype and phoR, wildtype and double mutant, sigB and phoR, sigB and double mutant, and phoR and double mutant. Any gene with an absolute rank difference below the threshold *a* for all six comparisons across all four time points is included in the self-consistent set. These stringent criteria allow only a small, conservative set of genes to be deemed self-consistent. The size of this set is largely determined by the parameter *a*, the maximum absolute rank difference allowed. Once the SCS set is identified, each gene on a given array is divided by the total expression of the SCS set on that array to normalise the data. Genes that show a consistently high or low expression may skew the SCS and introduce bias, therefore a number of genes were excluded before the filter stage by finding the average of the contributions for each gene on all arrays and excluding the top and bottom *x*%.

### 2.2. Sensitivity of SCS normalisation to parameter settings

**Figure 2:**
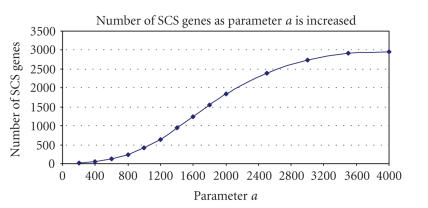
Number of genes identified as SCS with increasing value of a. The maximum number of possible SCS genes is 2958 as genes that are unreliable or have zero expression are excluded from the SCS during the implementation of the algorithm.

**Figure 3:**
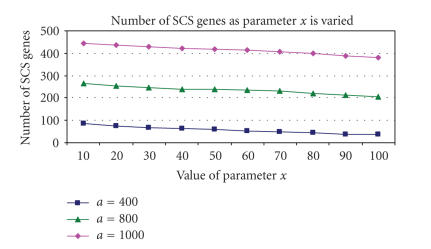
Effect of parameter *x* on SCS size. Influence of parameter *x* (the percentage of genes excluded prior to identifying SCS genes) over the number of SCS genes identified for different values of parameter *a*.

**Figure 4:**
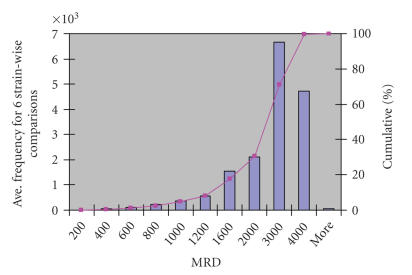
Minimum rank difference (MRD) histogram. MRD is calculated for each gene and each of the six strain-wise comparisons.

**Figure 5:**
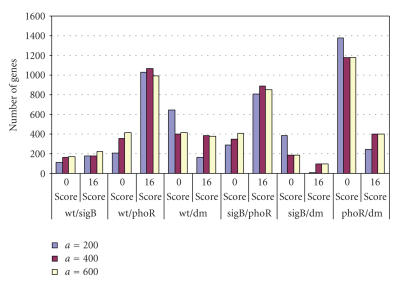
Extreme Park scores for each strain-wise comparison. The number of genes scoring either 0 or 16 when the Park score is calculated for each strain-wise comparison for different values of parameter *a*.

The number of SCS genes identified by the algorithm is largely dependant on the two user-defined parameters, *a*, the absolute rank difference limit, and *x*, the proportion of genes excluded. Figures 2 and 3 show how the number of genes identified as SCS changes depending on the values of *a* and *x*. It is clear that parameter *a* has a greater influence over the size of the SCS than *x*. As *a* increases so does the number of genes which pass the filters and end up in the final SCS whereas when *x* is increased, the stringency of the algorithm is increased as there are less potential SCS genes to start with. A wide range of *a* and *x* was investigated in this case to establish the sensitivity of the algorithm to these values, although it is clear that excessively large values of *a* result in an unrealistically large SCS gene sets. Several values of *a*, set to low proportion of the total number of genes spotted on the array, can be tested rapidly and the appropriate threshold selected (as described in this manuscript). Alternatively, a minimum ranking difference (MRD = min{rank(*i*) − rank(*j*)}) can be calculated for each gene and each condition. Figure 4 shows a histogram of MRD over all six comparisons performed in this study. The MRD distribution can then be used to estimate the threshold value of *a*. In this case, a comparison between sets obtained when parameter *a* values were set to 200, 400, and 600 (representing approximately 5%, 10%, and 15% of the total number of genes, resp.) was performed using the improvements in the number of differentially expressed genes (only data for genes scoring an extreme Park score of either 0 or 16 is shown here). The bar chart in Figure 5 shows that there is no detectable improvement in using *a* = 600 (resulting in 132 SCS genes). There is a more notable difference in the number of genes with an extreme Park scores when *a* = 200, however this only results in 11 SCS genes, which is a rather low proportion of the total number of genes spotted on the array. Thus the value of *a* = 400 (63 SCS genes) was chosen for the future analysis of this data set.

**Figure 6:**
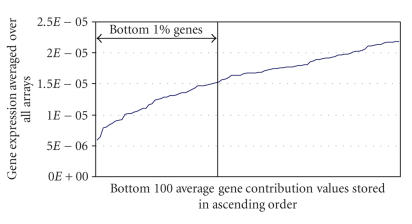
Lower end of gene contribution values averaged over all arrays. The average contribution values of the bottom 100 genes when the average contributions data is sorted in ascending order. When parameter *x* is set to 1%, the 40 genes with the lowest average contributions are excluded from the SCS.

**Figure 7:**
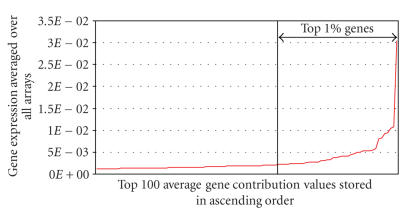
Upper end of gene contribution values averaged over all arrays. The average contribution values of the top 100 genes when the average contributions data is sorted in ascending order. When parameter *x* is set to 1%, the 40 genes with the highest average contributions are excluded from the SCS.

Parameter *x* was varied from 0.5%–5% of the total number of genes in the data set, for three different values of *a* (Figure 3). The number of SCS genes decreases linearly as *x* increases. Parameter *x* was selected to exclude 40 genes (∼1% of the total number of genes spotted on the array) as shown in Figures 6 and 7. This results in a reduction in standard deviation of 62% on average across all the arrays.

The application of the SCS algorithm, using *a* = 400 and *x* = 1%, to the full *B. subtilis* data set resulted in 63 SCS genes (2% of the total number of genes spotted on the array). These genes are listed in Table 1, grouped into functional categories as defined in SubtiList Web Server [12]. The raw expression values for these genes vary over the time course of the experiments, however the rank positions of the contributions of these genes are similar for any given time point in each experiment. The SCS set represents genes whose expression remains relatively unchanged under the experimental conditions investigated here.

### 2.3. Noise reduction

**Figure 8:**
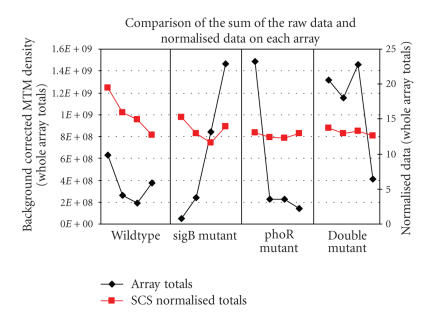
Raw gene expression and normalised data totals. Comparison of the raw (nonnormalised) background gene expression array total (black diamonds) and SCS normalised data (red squares) using values of *a* = 400, *x* = 1%.

Although the lack of technical replicates precludes the use of coefficient of variation as a measure of noise reduction, the analysis of totals of gene expression for each array can serve as a useful tool to assess the impact of the normalisation procedure. Figure 8 shows a comparison of the totals of the raw gene expression for each array (black diamonds) as well as the SCS normalised totals (red squares). A standard global scaling normalisation, such as normalised median-based trimmed mean (nMTM) density—an output of the array image analysis software ArrayVision, would simply result in a straight line of totals for all the arrays since it assumes linear relationship between the intensities on different arrays and forces the total intensity on each array to be equal. This is an assumption which only holds true if a small number of genes are expected to change between conditions. Biologically, we are expecting to see a reduction in overall gene expression over the time course in each strain as phosphate starvation is encountered. Clearly the variations between array totals are significantly reduced in the case of SCS normalised data as shown in Figure 8. In addition, between-array MA plots of normalised data (both time-point and strain-wise comparisons, not shown here) revealed no block effects or introduction of artefacts by the normalisation technique.

### 2.4. Differential gene expression

**Figure 9:**
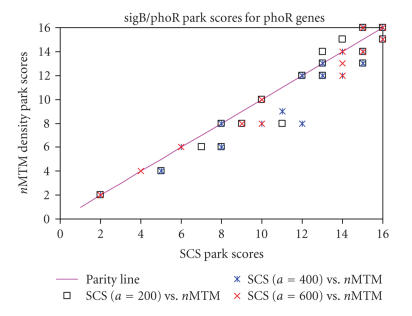
Park scores for sigB/phoR comparison. Comparison of the Park scores for the 33 known genes under the pho regulon control in the *sigB*-null mutant versus *phoR*-null mutant using globally scaled normalisation (nMTM) and SCS-normalised data. Park scores are shown for the SCS normalisation when parameter *a* is set to 200, 400, and 600. Note that some of the symbols are placed in identical positions when the Park scores are identical for both data sets.

**Figure 10:**
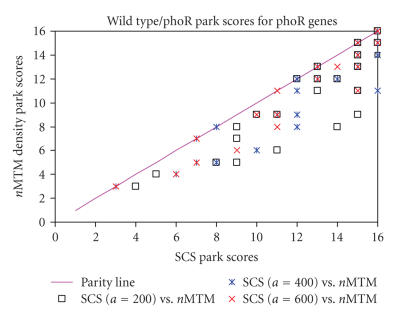
Park scores for wild type/phoR comparison. Comparison of the Park scores for the 33 known genes under the pho regulon control in the wildtype strain versus *phoR*-null mutant using globally scaled normalisation (nMTM) and SCS-normalised data. Park scores are shown for the SCS normalisation when parameter *a* is set to 200, 400, and 600. Note that some of the symbols are placed in identical positions when the Park scores are identical for both data sets.

In the absence of technical replicates in the data set studied, it has proved difficult to apply the common methodologies employed to identify differentially expressed genes such as the *t*-statistic or Wilcoxon test. Instead, each gene's Park score has been calculated for every strain-wise comparison for both the nMTM- and SCS-normalised data sets. There is variation in the Park scores between the two normalisation methods and the results indicate differentially expressed genes are more likely to be correctly identified when the data is normalised with the SCS method rather than the nMTM. To illustrate this, a subset of 33 genes known to be under the control of *phoR* is focussed on since the expression of the pho-regulated genes is expected to be notably lower in the *phoR*-null mutant compared to the wildtype or the *sigB*-null mutant. Therefore the Park scores for the wildtype/phoR and the sigB/phoR comparisons are shown (Figures 9 and 10). These 33 genes (with the exception a small number of genes in the pho regulon that are repressed by phoR) are expected to have a high Park score in the two comparisons, indicating that they are expressed to a greater degree in the wildtype or sigB strain compared to the phoR strain.

The parity plots (Figures 9 and 10) show the Park score for the selected genes using the two normalisation tech-niques. With the SCS normalisation, the Park scores for different values of parameter *a* (200, 400, and 600) are also shown. If the two techniques were equal, all the symbols are expected to lie on the parity line. Of the 33 pho-regulated genes shown in Figure 9 (the sigB/phoR comparison) 58% have a higher Park score with the SCS normalisation (*a* = 400), so that the points lie below the parity line. A further 36% have equal Park scores when under both normalisation techniques and only 3 genes have a higher Park score when normalised using the global scaling method. No major improvement is seen when parameter *a* is set to 200 or 600. A similar result is seen in Figure 10 (the wildtype/phoR comparison). In this case 42% of the pho-regulated genes have equal Park scores in both normalisation techniques and the remaining 58% have a higher Park score with the SCS normalisation (*a* = 400). This indicates that the SCS normalisation allows a clearer discrimination of the genes which are known to be differentially expressed in this experimental system.

## 3. DISCUSSION

The proposed normalisation technique results in a set of genes deemed to be self-consistent. These genes show a level of consistency throughout the whole data set based on ranked positions on each array. Using rank positions rather than actual expression or contribution data means that whole array effects, such as exposure length, may be disregarded as we can assume that they will not affect the rank positions of the gene expression contributions. The resulting SCS is a small conservative set of genes. Some of these genes will have a stable level of expression in all experimental conditions and may be classed as perhaps core genes or essential to the cell's basic functions. When this SCS list is compared to the list of essential *B. subtilis* genes published by Kobayashi et al. [13], we find that a small number of the SCS genes are indeed essential for the organism's survival. In particular *dnaE*, *ftsZ*, *pgk*, *rplD*, *rplJ,* and *yurV* appear on both lists. There are also a number of other genes on the SCS list that may belong to the same operons or pathways as some of the essential *B. subtilis* genes. In total, about a third of our SCS genes appear to be essential or linked to essential genes, however the list of essential genes was generated by growing *B. subtilis* in optimum conditions. Therefore these genes may behave differently in the phosphate-limited conditions or in the mutant strains used in this experiment. For instance, *tagA*, *B*, *D*, and *F* are listed as essential genes, but are also under the control of *phoR* which is knocked out in the *phoR*-null mutant [14], likewise *nadE* and *spoVC* are linked with the *sigB* gene [15, 16], the genes knocked out in the *sigB*-null mutant.

Results from synthetic data set analyses [17] confirm that the algorithm indeed identifies a suitable set of SCS genes on the basis of underlying biological signal rather than chance correlation in the expression data.

### 3.1. Parameter setting

Whilst the authors believe that the SCS normalisation algorithm is generally applicable to any three-dimensional experimental data as shown in Figure 1, it is essential that the values of parameters *a* and *x* are selected appropriately for each data set. This can be performed in a straightforward manner by repeating the SCS normalisation procedure with a number of combinations of these parameters and the resulting SCS gene sets evaluated as shown here, with limited computational effort. Alternatively, statistical methods can be applied to investigate the probability behaviour of minimum rank difference (MRD) distribution and to estimate the threshold value of *a*.

### 3.2. Noise reduction: totals

Figure 8 shows that the variability of the normalised data is much lower than that of the raw MTM density data. The change in total expression over time is biologically more plausible when the data is normalised using this algorithm. The three mutants initially have a lower total gene expression than the wildtype strain. The phoR strain and the double mutant show a similar pattern of total expression over time compared to the wildtype and the sigB strain. This can be expected as the organism does not have the mechanism to specifically cope with phosphate stress in the *phoR-null* mutant or the double mutant. The wildtype strain shows decrease in total gene expression over time when the data is normalised. It is expected that under phosphate-limited conditions the organism will eventually sporulate and so will downregulate a number of metabolic pathways, hence reducing the overall amount of mRNA in the cells. The sigB strain also shows a decrease in total gene expression, when the data is normalised, up to the last time point, where the total gene expression increases. A biological explanation for this could be related to the hyperinduction of the phoR operon or the onset of sporulation. Upon inspection it transpires that 124 genes are upregulated by at least 3 fold between the last two time points of the SCS-normalised data in the *sigB-null* mutant. Of these genes 32% are either related to sporulation or involved in reaction pathways that result in the release of phosphate. A further 37% of these up-regulated genes currently have an unknown function. The remaining 31% have varying functions but mainly belonging to functional categories 1 and 2 (see SubtiList [12] for functional category classification). In the three mutant strains, overall gene expression was lower at the outset (as indicated in Figure 8) and confirmed by independent assays [18]. This is clearly not the case with the nonnormalised data, where the total gene expression in the phoR strain and the double mutant is relatively high at the outset (shown by the black markers in Figure 8).

### 3.3. Differentially expressed genes

Although the lack of technical replicates precluded the application of a number of standard statistical tests as reported in the literature (e.g., *t*-tests and Wilcoxon test), The Park score analysis of these genes clearly shows that SCS normalisation enables better discrimination of the differentially expressed genes in different mutant strains (Figures 9 and 10).

## 4. CONCLUSIONS

A nonparametric normalisation method is proposed for multicondition time series gene expression data. This method is based on a series of comparisons of ranked gene expression contributions on the individual arrays. If the rank position of a gene contribution, to the array total, does not change within specified limits across all the arrays then that gene is included in the self-consistent set (SCS) of genes. The total expression of these genes on each of the arrays is then used to normalise the expression data of the rest of the genes. The algorithm depends upon two user-defined parameters, *a*, the absolute rank difference limit and, to a lesser degree, *x*, the proportion of genes excluded. The results of simulated studies using randomly generated synthetic data sets [17] confirmed that the SCS normalisation performs as expected. Current work concentrates on robustness studies of the SCS normalisation in order to assess the sensitivity of the algorithm to experimental data corrupted by known random and systematic noise. Also the application of this method to other gene expression data containing a number of technical replicates, which exhibits the same structure shown in this manuscript, is being investigated.

We believe that the proposed normalisation method may be useful in other cases of single colour DNA array analysis with a combination of multiple strains, conditions, and/or time points. The method provides a way of normalising using all the data simultaneously without having to assign a baseline array or using complex statistics that require replicate data. Using this approach will allow us to apply further data analysis techniques with more confidence in the biological plausibility of the results. Therefore the time, money and effort that has been put into producing this data set in the first place will not be entirely lost due to oversight of the importance of technical and biological replication and therefore some useful knowledge may still be gained from the data.

## 5. METHODS

### 5.1. Array dataset

Data has been obtained from experiments where both the specific and nonspecific response to phosphate stress has been investigated in a set of isogenic *Bacillus subtilis* mutants over time [18]. The overall aim was to identify regulatory interactions between the *σ^B^*-dependent general stress and pho regulons in *B. subtilis*. Strains with null mutations in the key regulatory genes *sigB* and *phoR* were used to investigate the level of interaction between these two regulons. In total, four strains were used: a wildtype strain (strain 168), *sigB-null *mutant, *phoR-null *mutant, and a *sigB-null, phoR-null* (double) mutant. For a detailed description of the bacterial strains, plasmids, primers, and medium used, see Allenby et al. [19]. Each strain was cultured in phosphate-limiting conditions with typically four samples taken at specified times. These samples were processed and used in transcriptome analysis by hybridising to *B. subtilis* Panorama gene arrays (Sigma Genosys Biotechnologies Inc., The Woodlands, USA). The procedures of cell harvesting, RNA preparation, synthesis of radioactively labelled cDNA, and hybridisation to the arrays as described by [20] were followed. Arrays were exposed on a Fuji cassette for a predetermined time. After exposure, the cassette was scanned using a Storm phosphorimager to generate both .gel and .tiff image files. These digital images were imported into the software package ArrayVision to generate the data set.

### 5.2. Use of technical replicates

The replicated spots on the arrays were used to identify any poor quality spots. The log_2_ transform of the data was used and for each array the difference of the two spots was plotted against the average of the two spots. The variability of the differences as a function of average intensity can be modelled using a locally smoothed estimate of the interquartile range. It has been shown that unreliable replicates can be identified by plotting lines representing ±3 × IQR on the MA plot [21]. Any replicate pair falling outside these lines can be deemed to have a replicate difference greater then expected based on their average value. Any gene identified as having poor replicates on any array was flagged and kept out of the self-consistent set during the normalisation algorithm.

### 5.3. SCS algorithm

**Figure 11:**
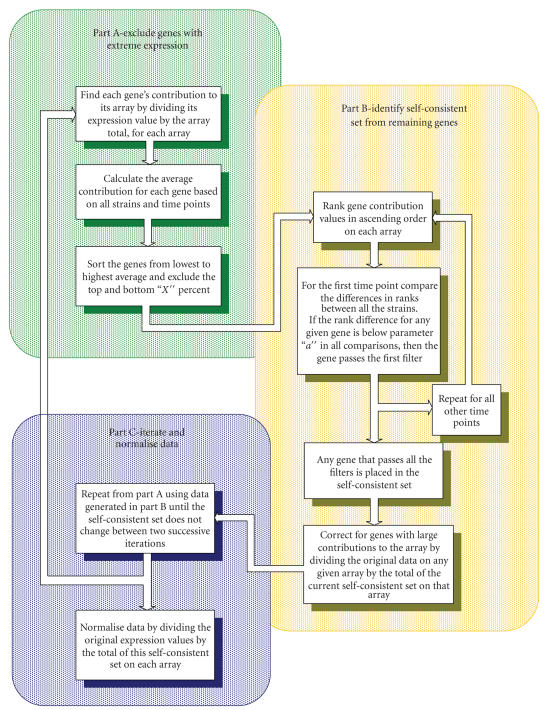
Flow Diagram of SCS algorithm. SCS algorithm depicted as a flow diagram showing the three stages of the algorithm.

Below is a mathematical description of the SCS algorithm, Figure 11 describes the process as a flow diagram.

For a (*m × n*) data set where *m* is the number of genes (rows) and *n* is the number of arrays (columns), each element of the data set is *g_ij_*, where *i* = 1 to *m* and *j* = 1 to *n*. For multistrain time series data *n = s* × *t*, where *s* = the number of strains from 1 to *S* and *t* = the number of time points from 1 to *T*.

First, the contributions matrix *C* is generated by dividing each gene expression value by the column total

(1) $${\left(c_{i}=\frac{g_{i}}{\sum_{i=1}^{m}g_{i}}\right)}_{j}$$.

The average of each row of contributions is calculated and the top and bottom *x*% is disregarded

(2) $$R=\left\{mx<\text{rank}{\left(\frac{\sum_{j=1}^{n}c_{j}}{n}\right)}_{i}<\left(m-mx\right)\right\}$$,

*R* is a vector of row numbers left once the top and bottom *x*% have been excluded. These row numbers are used to generate a new contributions matrix *C*_2_ which is a subset of the matrix *C*. It is from this new (*m* − 2*mx*) × *n* matrix that the initial SCS genes will be identified

(3) $$C_{2}=\left(\forall R\right)C_{2}\subseteq C$$.

For time point *t*,

(4) SCS*_t_* = {( | rank (*C*_2(*s*1)_) − rank (*C*_2(*s*2)_) | )*_t_* < a&
( | rank (*C*_2(*s*1)_) − rank (*C*_2(*s*3)_) | )*_t_* < a&
( | rank (*C*_2(*s*2)_) − rank (*C*_2(*s*3)_) | )*_t_* < a}.

In (4), shown here there are three strains to consider, as the rank differences are calculated for each possible pairing of strains, the more strains there are, the more terms are needed in the equation.

This is carried out for each time point to give SCS_1_, SCS_2_, SCS_3_, …, SCS*_T_*. Then any gene that appears in all the SCS*_t_* lists is deemed to be self-consistent across all strains and time points. These genes are then used to normalise the data by dividing each column of data by the sum of the SCS genes in that column

(5) $${\left(N_{i}=\frac{g_{i}}{\sum gscs}\right)}_{j}$$.

The process is then iterated *k* times by repeating each step from the calculation of the contributions until no change is seen between SCS*_k_* and SCS_*k*−1_.

### 5.4. Differential expression of genes

**Figure 12:**
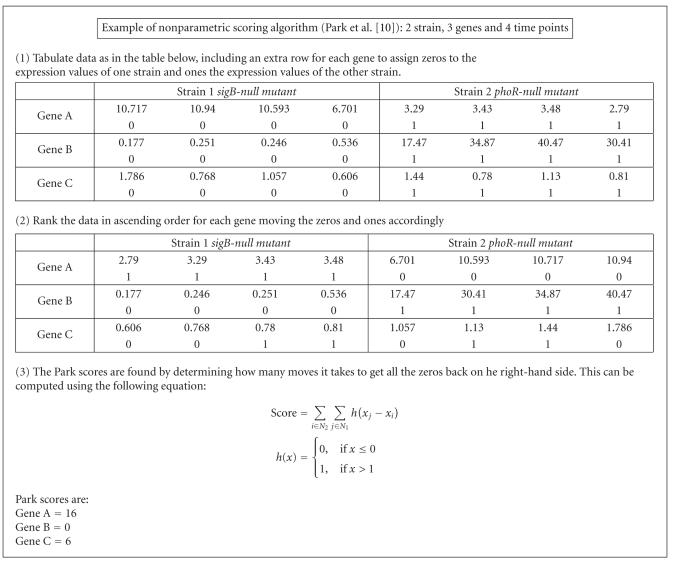
Example of Park score calculations. Park score calculations shown for the expression data of three genes in two different strains over four time points. Genes A and B score an extreme Park score as they are expressed more highly at every time point in one of the strains compared to the other strain, whereas Gene C is not and scores a midrange Park score.

A nonparametric Park score test [10] was also used to assess the differential expression of the same genes in the same two strains using the globally scaled nMTM density data from ArrayVision and SCS-normalised data. Figure 12 shows the basic features of the method. For the Park score analysis, data from the *sigB-null *mutant was entered as “strain1” and data from *phoR-null *mutant as “strain2” data.

### 5.5. Application to data sets

Before the SCS normalisation (see detailed description of the algorithm in Section 5.3) was applied to the *B. subtilis* data set, unreliable replicates were flagged and any genes with an expression value below that of the array background value were also flagged. The genes falling below the background values were taken as having an expression too low to accurately detect and were forced to have an expression value of zero. These flagged genes were prevented from being part of the SCS but were not excluded from the data set at this point. Different values of the absolute rank difference threshold, *a*, and the exclusion limit, *x*, were tested and the resulting SCS gene sets recorded and analysed.

## Figures and Tables

**Table 1:** Functions of the *63 SCS genes* identified in the *B. subtilis* data. The functional descriptions and functional categories were obtained from the SubtiList Web Server [12]. The 63 SCS genes were identified with parameter *a* = 400.

Gene name	Function*

*1. Cell envelope and cellular processes*

med	Positive regulator of comK
msmX	Multiple sugar-binding transport ATP-binding protein
nark	Nitrite extrusion protein
rocC	Amino acid permease
pstS	Phosphate ABC transporter (binding protein)
yfmO	Similar to multidrug-efflux transporter
ytlD	Similar to ABC transporter (permease)
ytrB	Similar to ABC transporter (ATP-binding protein)
yurO	Similar to multiple sugar-binding protein
yvfR	Similar to ABC transporter transmembrane subunit
yvfS	Similar to ABC transporter transmembrane subunit
ywoE	Similar to permease
yfiJ	Similar to two-component sensor histidine kinase [YfiK]
tatCY	Component of the twin-arginine translocation pathway
ftsZ	Cell-division initiation protein
phrA	Inhibitor of the activity of phosphatase RapA
ykuD	Similar to hypothetical proteins
comFB	Late competence gene

*2. Intermediary Metabolism*

ptsH	Histidine-containing phosphocarrier protein of the phosphotransferase system (PTS) (HPr protein)
pgk	Phosphoglycerate kinase
sdhA	Succinate dehydrogenase (flavoprotein subunit)
gcvPB	Probable glycine decarboxylase (subunit 2)
argH	Argininosuccinate lyase
trpD	Anthranilate phosphoribosyltransferase
pucA	Xanthine dehydrogenase
purC	Phosphoribosylaminoimidazole succinocarboxamide synthetase
yabR	Similar to polyribonucleotide nucleotidyltransferase
lipA	Probable lipoic acid synthetase
moaE	Molybdopterin converting factor (subunit 2)

*3. Information Pathways*

dnaE	DNA polymerase III (alpha subunit)
uvrA	Excinuclease ABC (subunit A)
sigL	RNA polymerase sigma factor
glcR	Transcriptional repressor involved in the expression of the phosphotransferase system
hpr	Transcriptional repressor of sporulation and extracellular proteases genes
lrpC	Transcriptional regulator (Lrp/AsnC family)
spoVT	Transcriptional regulator
yetL	Similar to transcriptional regulator (MarR family)
yisV	Similar to transcriptional regulator (GntR family)/aminotransferase (mocR-like)
rplD	Ribosomal protein L4
rplJ	Ribosomal protein L10 (BL5)
ykkC	Similar to chaperonin

*4. Other Functions*

yvtA	Similar to htrA-like serine protease
albC	Antilisterial bacteriocin (subtilosin) production
ppsB	Peptide synthetase
xtmA	PBSX defective prophage terminase (small subunit)
pcrB	pcrB homolog
yurV	Similar to NifU protein homolog

*5. Similar to Unknown Proteins*

ycgL	Similar to unknown proteins
ydiI	Similar to unknown proteins
yisX	Similar to unknown proteins
ykkA	Similar to unknown proteins
yrbG	Similar to hypothetical proteins from B. subtilis
yshB	Similar to unknown proteins
yutH	Similar to unknown proteins
ywnB	Similar to unknown proteins
yazA	Similar to unknown proteins
ybbP	Similar to unknown proteins
yfkC	Similar to unknown proteins
yloN	Similar to unknown proteins
ytqA	Similar to unknown proteins
yveS	Similar to unknown proteins

*6. No Similarity*

ybdL	Unknown
ydaS	Unknown
